# Galactomannan from *Trigonella foenum*‐*graecum* L. seed: Prebiotic application and its fermentation by the probiotic *Bacillus coagulans* strain MTCC 5856

**DOI:** 10.1002/fsn3.606

**Published:** 2018-02-20

**Authors:** Muhammed Majeed, Shaheen Majeed, Kalyanam Nagabhushanam, Sivakumar Arumugam, Sankaran Natarajan, Kirankumar Beede, Furqan Ali

**Affiliations:** ^1^ Sami Labs Limited Bangalore Karnataka India; ^2^ Sabinsa Corporation East Windsor NJ USA; ^3^ Sabinsa Corporation Payson UT USA

**Keywords:** *Bacillus coagulans*, fenugreek seeds, fermentation, galactomannan, synbiotic

## Abstract

Health benefits of dietary fibers are currently being widely recognized. However, the assessment of dietary fiber as a prebiotic is essential and also important for the development of an improved synbiotic commercial preparation. Thus, the aim of this study was to evaluate the potential of galactomannan extracted from fenugreek seeds as a prebiotic fiber and also its fermentation by the probiotic strain *Bacillus coagulans* MTCC 5856. Nondigestibility by the gastric acid and pancreatic enzyme hydrolysis of galactomannan were determined using an in vitro model mimicking the in vivo conditions. Further, anaerobic fermentation and utilization of galactomannan by the *B. coagulans* MTCC 5856 was investigated followed by selective inhibition of *Escherichia coli* ATCC 25922. The galactomannan from fenugreek seeds was found to be nondigestible to gastric acid and also to pancreatic enzymatic hydrolysis. The galactomannan was fermented and utilized (71.4%) by the *B. coagulans* MTCC 5856, and also significant amount of short‐chain fatty acids production was also observed. Furthermore, *B. coagulans* MTCC 5856 inhibited the *E. coli* ATCC 25922 growth when cocultured with galactomannan suggesting competitive fermentation of probiotic bacteria. Galactomannan exhibited prebiotic activity and also showed suitability with probiotic *B. coagulans* MTCC 5856 in a synbiotic combination. This study provides the first scientific evidence of galactomannan from fenugreek seeds as a prebiotic that may play an important role in modulating gut flora by acting as substrate to beneficial microbes.

## 1 INTRODUCTION

The term “prebiotics” was named by Gibson and Roberfroid in 1995 and defined as nondigestible fiber in the upper gastrointestinal tract which can enhance the growth or activity of advantageous bacteria of large bowel by acting as substrate for them (Gibson & Roberfroid, 1995). Further, Marcel Roberfroid in 2007 proposed that the prebiotics can be defined as “a selectively fermented ingredient that allows specific changes, both in the composition and/or activity in the gastrointestinal microflora that confers benefits upon host well‐being and health” (Roberfroid, 2007). Later, Hutkins et al. in 2016 defined prebiotics as “food ingredients that help support growth of probiotic bacteria” or “nondigestible substances that act as food for the gut microbiota” (Hutkins et al., 2016). There are prebiotic compounds such as galactooligosaccharide and inulin and the dietary fibers such as resistant starch, pectin, beta‐glucans, and xylooligosaccharides that qualify for the prebiotics definition (Linares‐Pastén, Aronsson, & Nordberg, 2017; Roberfroid, 2007; Zaman & Sarbini, 2015). Prebiotics confer the benefits by supporting the growth of probiotics in the colon by serving as a substrate. The end products of this fermentation are primarily short‐chain fatty acids (SCFAs) and other metabolites. The major SCFAs produced during prebiotics fermentation are acetic, propionic and butyric acids as end products (Pylkas, Juneja, & Slavin, 2005). Literature suggested that SCFAs have potential role in the human health as an energy source and also inhibiting the growth of pathogenic bacteria by decreasing the colonic pH (Pylkas et al., 2005).

*Trigonella foenum‐graecum* (fenugreek) is known for its traditional medicinal use to improve digestion and for the management of blood sugar levels (Srinivasan, 2005). Fenugreek seeds and leaves are the common food spices used in the Middle East and South Asia. Fenugreek seeds have been reported for various pharmacological activities such as hypocholesterolemic, immunological activity, lactation aid, antibacterial, gastric stimulant, for anorexia, antidiabetic agent, galactagogue, hepatoprotective effect, and anticancer activity (Murlidhar & Goswami, 2012). The main component of fenugreek seeds is galactomannan which is a polysaccharide structurally composed of a 1→4 beta‐D‐mannosyl backbone substituted by a single galactose unit α‐linked at the C‐6 oxygen (Jiang, Zhu, Zhang, & Sun, 2007). The soluble nature of galactomannan fiber from fenugreek has been linked to numerous human health benefits such as reduction in LDL cholesterol levels in hypercholesterolemic, blood lipids, blood pressure, and fibrinolysis in healthy men (Srinivasan, 2005). It also has been reported to significantly improve glucose homeostasis in type 1 and type 2 diabetes by delaying carbohydrate digestion and absorption (Hannan et al., 2007).

Probiotics are the “live microorganisms which, when administered in adequate amounts, confer a health benefit on the host” (FAO/WHO, 2002). *Bacillus coagulans* MTCC 5856 is a spore‐forming bacteria which is a commercial probiotic preparation sold under the trade name LactoSpore^®^ nearly for two decades (Majeed, Nagabhushanam, Natarajan, Sivakumar, Eshuis‐de Ruiter, et al., 2016). In a rodent model, *B. coagulans* MTCC 5856 exerted antidiarrheal activity and also inhibited the gastrointestinal motility at a dose of 160 × 10^6^ spores/kg body weight (Majeed, Natarajan, et al., 2016). In a placebo‐controlled human trial, *B. coagulans* MTCC 5856 was found to be safe and tolerable at a dose of 2 × 10^9^ spore/day when supplemented for 30 days (Majeed, Kalyanam, et al., 2016). Another controlled human clinical trial revealed that *B. coagulans* MTCC 5856 has demonstrated significant efficacy in patients with diarrhea‐predominant IBS in mitigating their clinical symptoms at a dose of 2 × 10^9^ spore/day when supplemented for 90 days (Majeed, Nagabhushanam, Natarajan, Sivakumar, Ali, et al., 2016). Further, *B. coagulans* MTCC 5856 was found to be highly stable during processing and storage of various functional foods due to its spore nature (Majeed, Majeed, et al., 2016) and also phenotypically and genotypically consistent over multiple years of commercial production (Majeed, Nagabhushanam, Natarajan, Sivakumar, Eshuis‐de Ruiter, et al., 2016).

Synbiotic formulation of probiotics with natural plant‐based fibers reported as a promising therapeutic and dietary approach (Bengmark & Martindale, 2005). The accomplishment of such an approach depends on careful selection of specific probiotic microorganisms whose viable count is effectively enhanced by natural plant‐based fibers, resistant to acid and enzymatic hydrolysis in the gut. These studies are critically important to accommodate the performance of host animals exposed to synbiotic diet regimes given that there are limitations to fiber digestion and utilization by microbes in terms of microbial accessibility to substrates, physical and chemical nature of fibers, and also kinetics of the digestive process (Varga & Kolver, 1997). Thus, this study was conducted to investigate the utilization of galactomannan from fenugreek seeds as prebiotics along with commercial probiotic strain *B. coagulans* MTCC 5856. This will provide important scientific evidence about the synbiotic formulation containing galactomannan from fenugreek seeds and the commercial probiotic strain *B. coagulans* MTCC 5856.

## 2 MATERIALS AND METHODS

De Man, Rogosa, and Sharpe (MRS) broth, potato soluble starch, glucose yeast extract agar, trypticase soy agar, eosin methylene blue agar (EMB Agar), glucose yeast extract agar (GYEA) were purchased from HiMedia, Mumbai, India. Lysozyme, pepsin, fructooligosaccharide (FOS), pancreatin from porcine, bile salt, copper sulfate, and SCFAs standards (acetic, propionic and butyric acids) were procured from Sigma‐Aldrich (St. Louis, MO, USA). Oxyrase was procured from Oxyrase, Inc, Mansfield, OH, USA. Probiotic bacteria *B. coagulans* MTCC 5856 is a patented strain of Sami Labs Limited and deposited to Microbial Type Culture Collection and Gene Bank (MTCC), Chandigarh, India. *E. coli* ATCC 25922 was obtained from American Type Culture Collection (ATCC, Manassas, VA, USA). Trypticase soy agar (TSA; Becton Dickinson) was used for culturing of bacteria, and culture stocks were maintained in aqueous glycerol (15%, v/v) at −80°C.

### 2.1 Method of preparing galactomannan from fenugreek seeds

Galactomannan was extracted from the fenugreek seeds as per the proprietary method (chemical and enzymatic) of Sabinsa Corporation, USA, by following current good manufacturing practices (cGMP). This commercial product is sold under the brand name Fenumannan^®^ which contains minimum 60% of galactomannan. The galactomannan content was determined using Megazyme kit as per the manufacturer's instructions (Megazyme International Ireland, Wicklow, Ireland). Carbohydrate content was quantified by Anthrone method using dextrose as standard (Trevelyan, Forrest, & Harrison, 1952). Protein content was estimated by Lowry's method using bovine serum albumin (Sigma‐Aldrich) as standard (Lowry, Rosebrough, Farr, & Randall, 1951). Total dietary fibers including soluble and insoluble were determined by enzymatic–gravimetric method as described in Association of Analytical Communities (AOAC <985.29>). Ash content and relative water solubility were measured by the method according to the United States Pharmacopeia (Chapter <281>; <1236>).

### 2.2 Gastric acid hydrolysis

Two grams of galactomannan from fenugreek seeds was dissolved in 100 ml of a sterile electrolyte solution (6.2 g/L NaCl, 2.2 g/L KCl, 0.22 g/L CaCl_2_, and 1.2 g/L NaHCO_3_) containing 0.01% lysozyme (Sigma‐Aldrich) and 0.3% pepsin (Sigma‐Aldrich). After dissolving the galactomannan from fenugreek seeds, pH was decreased to 1.5 by adding 1.0 mol/L HCl and incubated at 37°C with 100 rpm for 180 min. Samples were taken at 0, 30, 60, 90, 120, and 180 min. Fructooligosaccharide (FOS) was taken in the study as a reference to compare with galactomannan from fenugreek seeds. In addition, potato soluble starch (HiMedia) was taken as a control. The release of reducing sugar was measured according to the previously described method (Miller, 1959; Nilsson & Bjorck, 1998).

### 2.3 Enzymatic hydrolysis

Pancreatin (100 mg) from porcine pancreas (Sigma‐Aldrich) was dissolved in 100 ml of phosphate buffer (50 mM; pH 7.0). Further, galactomannan from fenugreek seeds (2 gm) was dissolved in above pancreatin solution and incubated at 37°C with 100 rpm for 180 min. Samples were taken at 0, 30, 60, 90, 120, and 180 min. FOS was taken in the study as a reference to compare with galactomannan from fenugreek seeds, and starch was also taken as a control. The release of reducing sugar was measured according to the previously described method (Miller, 1959; Oku, Tokunaga, & Hosoya, 1988).

### 2.4 Fermentation

A single isolated colony of *B. coagulans* MTCC 5856 was inoculated into MRS broth (HiMedia) and incubated at 37°C with 120 rpm for overnight. Galactomannan from fenugreek seeds alone (0.5%, 1.0%, and 2.0%, w/v), and along with MRS media (devoid of dextrose) (0.5%, 1.0%, and 2.0%, w/v), was prepared. MRS broth and MRS (devoid of dextrose) were prepared separately. Similarly, fructooligosaccharide (FOS) was taken in the study as a reference control to compare with galactomannan from fenugreek seeds. The final pH of all the media was adjusted to 7.0. Five percent of overnight grown *B. coagulans* MTCC 5856 culture was inoculated to all the flasks and incubated at 37°C with 100 rpm for 24 h. pH values at 0 h of incubation and after 24 h of fermentation were recorded. Samples were serially diluted in sterile saline, and the viable count was enumerated by plating on glucose yeast extract agar (HiMedia) at the 0 h and after 24 h of fermentation as per the previously described method (Majeed, Majeed, et al., 2016). The plates were incubated at 37°C for 48–72 h. Each analysis was performed in triplicate at two different occasions. Average mean of viable counts is expressed in log_10_ cfu/ml.

### 2.5 Production of short‐chain fatty acids

The in vitro fermentation with the *B. coagulans* MTCC 5856 was carried out by following the method described by McBurney and Thompson (1987) with some modifications. Briefly, 2.0 g of glucose or galactomannan from fenugreek seeds was added to 100 ml of demineralized water. The pH was adjusted to 7.0 ± 0.2 and autoclaved at 121°C for 20 min. After sterilization, oxygen‐reducing enzyme Oxyrase (Oxyrase^®^ for Broth) was added to each flask, to induce anaerobic conditions. Five percent of overnight grown *B. coagulans* MTCC 5856 culture was inoculated to all the flasks and incubated at 37°C with gently shaken for 24 h. The bottles were tightly closed and sealed with parafilm to maintain anaerobic conditions generated by the enzyme supplement. The pH values at 0 h of incubation and after 24 h of fermentation were also recorded. One ml of copper sulfate (10 g/L) was added to each sample to inhibit further microbial growth (Sigma‐Aldrich). Further, 5.0 ml of samples was added to 5 ml of distilled water, and pH was adjusted to 1.5 using 3mol/L H_2_SO_4_. Chilled (−20°C) diethyl ether 10 ml was added to the samples and then mixed in a vortex mixer for 1 min. Sodium chloride was added and then centrifuged at 3000 ***g*** for 10 min. After centrifugation, organic layer was separated and transferred to the fresh vial. This was used to quantify SCFAs. The SCFA standards were purchased from Sigma‐Aldrich and similarly processed. SCFA production (acetic, propionic and butyric acids) was measured by gas chromatography (GC) with the use of a Agilent technologies 6890N gas chromatograph (Stevens Creek Blvd Santa Clara, CA, USA) containing a DB‐FFAP (terephthalic acid modified polyethylene glycol) column. The column temperature was 200°C. The injector and detector port temperatures were 250°C. The carrier gas was N_2_ at a flow rate of 1.0 ml/min. SCFA standards were purchased from Sigma‐Aldrich. SCFA (acetic, propionic and butyric acids) concentrations were expressed in mg/g of galactomannan.

### 2.6 Inhibition of *E. coli* ATCC 25922 growth

The in vitro experiment was designed to evaluate the effect of galactomannan from fenugreek seeds with probiotic bacteria *B. coagulans* MTCC 5856 for the inhibition of Gram‐negative pathogenic bacteria *E. coli* ATCC 25922. Briefly, 2.0 g of galactomannan from fenugreek seeds was added to 100 ml of demineralized water. The pH was adjusted to 7.0 ± 0.2 and autoclaved at 121°C for 20 min. After sterilization, oxygen‐reducing enzyme Oxyrase (Oxyrase^®^) was added to each flask. *Bacillus coagulans* MTCC 5856 was grown on glucose yeast extract agar (HiMedia), and *E. coli* ATCC 25922 was grown on trypticase soy agar (HiMedia). A single isolated colony of both the cultures was used, and the turbidity of the bacterial suspension was adjusted to 0.5 McFarland standards (equivalent to 1.5 × 10^8^ cfu/ml). One ml of *E. coli* ATCC 25922 was added to flask containing galactomannan from fenugreek seeds. Similarly, in other groups, one ml of *E. coli* ATCC 25922 and one ml of *B. coagulans* MTCC 5856 were added to flask containing galactomannan from fenugreek seeds. The flasks were incubated at 37°C with 100 rpm for 24 h. Samples were serially diluted in sterile saline, and the viable count of *E. coli* ATCC 25922 was enumerated by plating on eosin methylene blue agar (EMB Agar; HiMedia) at 0 h and after 24 h of fermentation. The plates were incubated at 37°C for 48 h. Each analysis was performed in triplicate at two different occasions. Average mean of viable counts is expressed in log_10_ cfu/ml.

### 2.7 Statistical analysis

All experiments were carried out in triplicates in at least three different occasions, and viable count of *B. coagulans* MTCC 5856 and *E. coli* ATCC 25922 was expressed in log_10_ cfu. Differences between two means were evaluated by the Student's *t* test. The chosen level of significance for all statistical tests was 5% (*p *<* *.05).

## 3 RESULTS

### 3.1 Characteristics of galactomannan from fenugreek seeds

The galactomannan content in the final product (Fenumannan^®^) was 63.52% (Table 1). The content of carbohydrate and protein in Fenumannan^®^ was 75.24% and 14.83%, respectively. The total carbohydrates content in Fenumannan^®^ included soluble fibers (65.42%), insoluble fibers (5.41%), and other carbohydrates. Estimation of galactomannan content revealed that it was mostly the water‐soluble fibers in Fenumannan^®^ (Table 1).

**Table 1:** Characteristics of galactomannan from fenugreek seeds

S. No.	Parameters	Content (%, w/w)
1	Soluble dietary fibers	65.42 ± 1.2
2	Insoluble dietary fibers	5.41 ± 0.41
3	Total dietary fibers	75.24 ± 1.2
4	Galactomannan	63.52 ± 2.1
5	Total carbohydrate	78.21 ± 2.2
6	Protein	14.83 ± 0.56
7	Ash	4.21 ± 0.16
8	Water soluble (1%, w/v solution in water)	82.75 ± 2.5

### 3.2 Gastric acid digestibility

**Figure 1:**
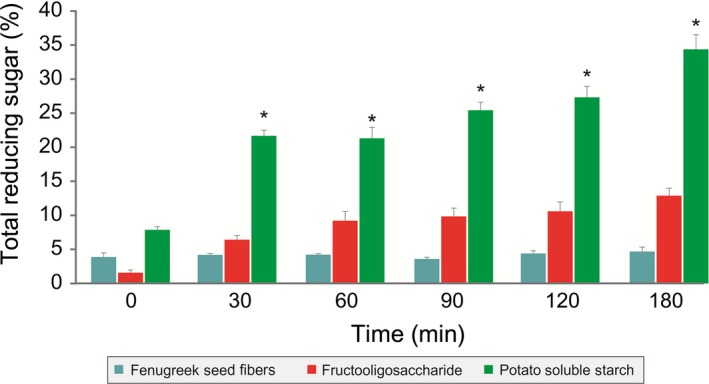
Effect of gastric acid hydrolysis on the galactomannan from fenugreek seeds. Potato starch and FOS showed significant increase in the reducing sugars after 180 min of gastric acid treatment compared to initial content (*p *<* *.05). Each value is the mean ± SD (*n* = 3). *p <* *.05; (Student's *t* test)

Nondigestibility of galactomannan from fenugreek seeds to the gastric acid was evaluated in an in vitro model mimicking the in vivo conditions. Three hours of gastric acid treatment to the galactomannan from fenugreek seeds did not result in the significant increase in reducing sugars thereby suggesting its nondigestibility to gastric acid (Figure 1). However, there was significant increase in the reducing sugars in the potato soluble starch and slight increase in FOS group (Figure 1).

### 3.3 Pancreatic enzyme digestibility of galactomannan from fenugreek seeds

**Figure 2:**
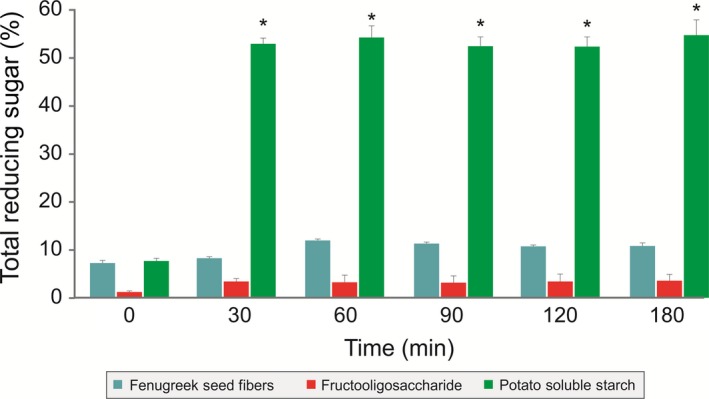
Effect of enzymatic digestibility on the galactomannan from fenugreek seeds. Potato starch used as control in the experiment showed significant increase in the reducing sugars after 180 min of gastric acid treatment compared to initial content (*p *<* *.05). Each value is the mean ± *SD* (*n* = 3). *p <* *.05; (Student's *t* test)

The enzymatic digestibility of galactomannan from fenugreek seeds was determined by treating galactomannan from fenugreek seeds with pancreatic enzymes for 3 h and measuring the reducing sugars at different time intervals. There was no significant change in reducing sugars in the galactomannan from fenugreek seeds and FOS group, thus indicating their enzymatic nondigestibility. However, a significant increase in reducing sugars in the potato soluble starch group was observed (Figure 2).

### 3.4 Fermentation by *B. coagulans* MTCC 5856

**Figure 3:**
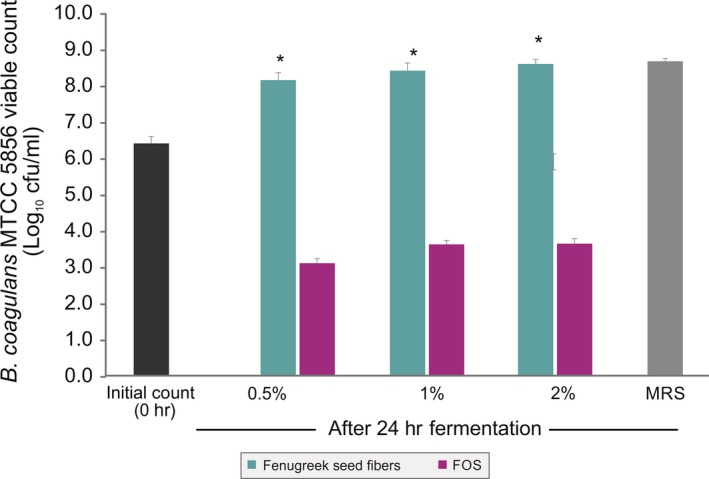
Effect of the galactomannan from fenugreek seeds as sole nutritional source on the viable count of *Bacillus coagulans* MTCC 5856. Different concentrations (0.5%, 1.0%, and 2.0%, w/v) of the galactomannan from fenugreek seeds were studied. *Bacillus coagulans* MTCC 5856 showed significantly higher viable count in fenugreek seed fibers compared to FOS (*p *<* *.05). Values are average mean of triplicate performed at two different occasions and represented in log_10_ cfu/ml. *p <* *.05; (Student's *t* test)

**Figure 4:**
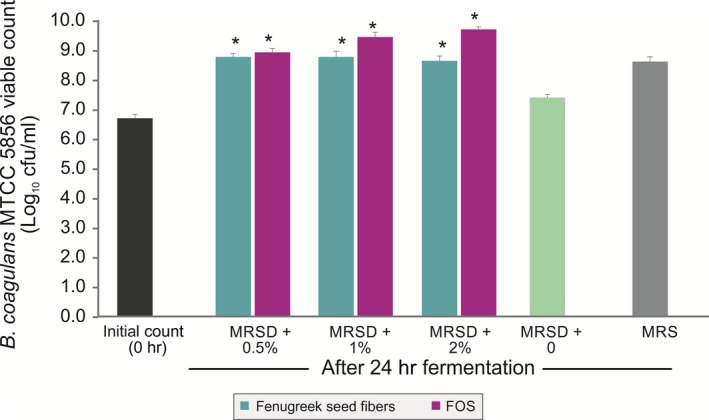
Effect of the galactomannan from fenugreek seeds along with other nutrients on the viable count of *Bacillus coagulans* MTCC 5856. MRSD is the MRS media devoid of dextrose. Different concentrations (0.5%, 1.0%, and 2.0%, w/v) of the galactomannan from fenugreek seeds were studied. *B. coagulans* MTCC 5856 showed significantly higher viable count in fenugreek seed fibers and FOS compared MRS media (*p *<* *.05). Values are average mean of triplicate performed at two different occasions and represented in log_10_ cfu/ml

**Figure 5:**
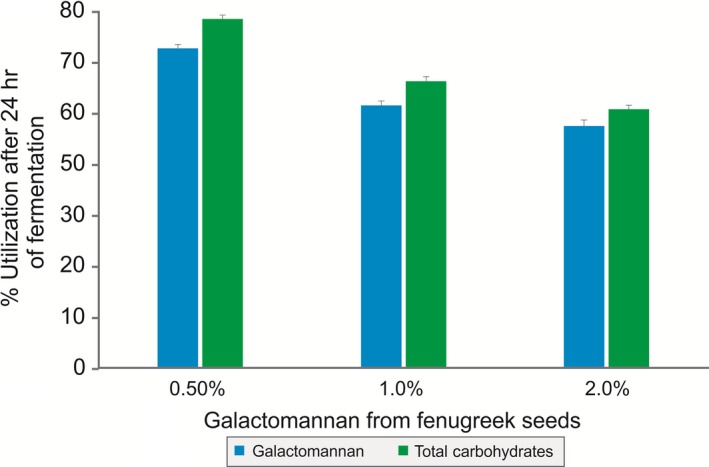
Utilization of galactomannan and total carbohydrate from fenugreek seeds after the anaerobic fermentation by *Bacillus coagulans* MTCC 5856. Each value is the mean ± *SD* (*n* = 3)

As galactomannan from fenugreek seeds contained dietary fibers and protein, it was used as a sole nutritional source and the growth of *B. coagulans* MTCC 5856 was determined by enumerating the viable count. *B. coagulans* MTCC 5856 was able to grow by utilizing galactomannan from fenugreek seeds, and there was 1.9 log_10_ cfu/ml increase (Figure 3) in the viable count after 24 h of fermentation (*p *<* *.05). The viable count of *B. coagulans* MTCC 5856 was similar in the galactomannan from fenugreek seeds group when compared with MRS media (*p *>* *.05). Further, galactomannan from fenugreek seeds supported the *B. coagulans* MTCC 5856 growth better as sole source of nutrition compared to FOS (*p *<* *.05). In another set of experiment, galactomannan from fenugreek seeds was used to replace carbon source (dextrose) in the enriched media (MRS). Galactomannan from fenugreek seeds (2.0%) group had slightly better *B. coagulans* MTCC 5856 count (0.2 log_10_ cfu/ml) compared to dextrose group (2.0%). However, FOS group (2.0%) showed better *B. coagulans* MTCC 5856 count (1.1 and 0.9 log_10_ cfu/ml) compared to dextrose (2.0%) and galactomannan from fenugreek seeds group (2.0%), respectively (Figure 4). Galactomannan utilization by *B. coagulans* MTCC 5856 was concentration dependent, and up to 71.4%, utilization was observed at a concentration of 0.5% (w/v) (Figure 5).

### 3.5 Production of SCFAs

**Figure 6:**
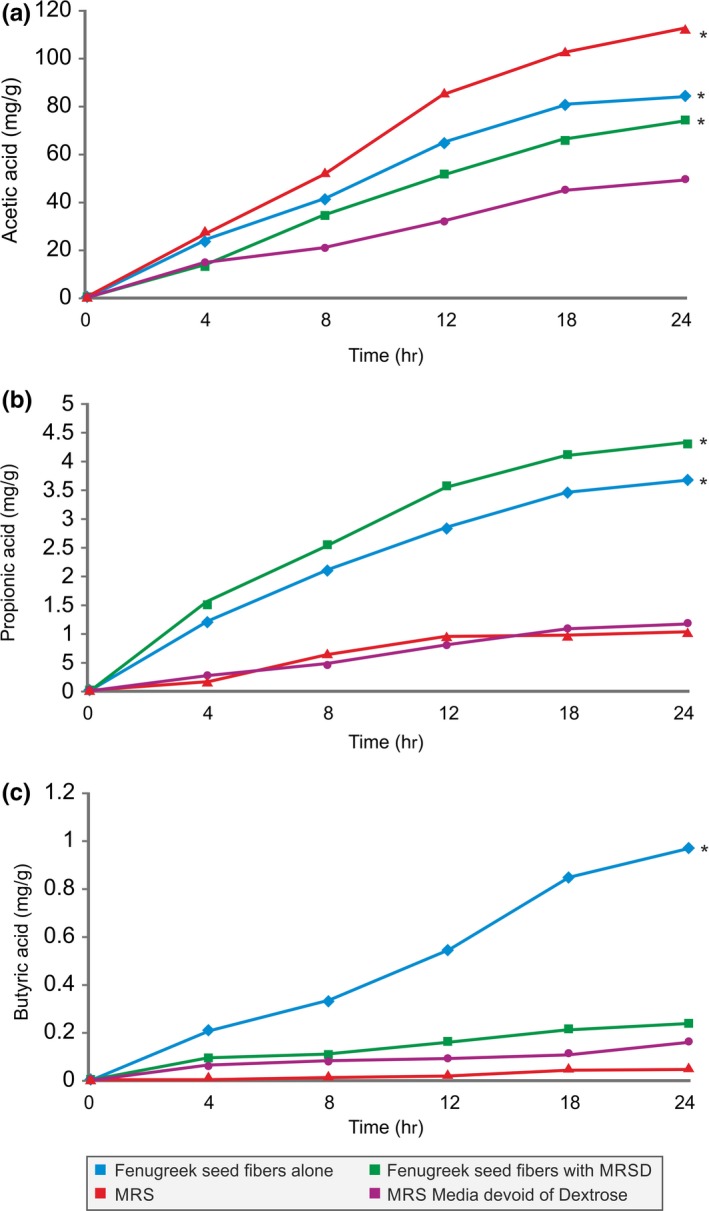
Acetic (a), propionic (b), and butyric acid (c) production from the galactomannan from fenugreek seeds alone and along with MRSD (MRS media devoid of dextrose), MRS, and MRS media devoid of dextrose after 0, 4, 8, 12, and 24 h in vitro batch culture fermentation with *Bacillus coagulans* MTCC 5856. Production of acetic acid (6A), propionic acid (6B), and butyric acid (6C) by the *B. coagulans* MTCC 5856 was significantly higher in fenugreek seed fibers compared to MRSD media devoid of dextrose (*p *<* *.05) after 24 h of incubation. Values are average mean of triplicate performed at two different occasions and represented in mg/g

The production of short‐chain fatty acids (acetic, propionic and butyric) by the *B. coagulans* MTCC 5856 by fermenting galactomannan from fenugreek seeds was studied. The production of acetic acid was highest (116 mg/g) among others when *B. coagulans* MTCC 5856 was grown in the dextrose group. However, the production was notable with only galactomannan from fenugreek seeds (82 mg/g) and with MRS (devoid of dextrose) plus galactomannan from fenugreek seeds (Figure 6a). Butyric acid production was highest (1 mg/g) in the galactomannan group followed by galactomannan with MRSD (MRS media devoid of dextrose) (Figure 6b). Similarly, propionic acid was highest in galactomannan (4.5 mg/g) and with MRSD (3.5 mg/g) (MRS media devoid of dextrose) followed by galactomannan group (Figure 6c). There was a time‐dependent short‐chain fatty acid production in all the groups, and highest production of acetic acid was observed followed by propionic acid and butyric acid, respectively, in all the groups (Figure 6a–c).

### 3.6 Inhibition of *E. coli* ATCC 25922 growth

**Figure 7:**
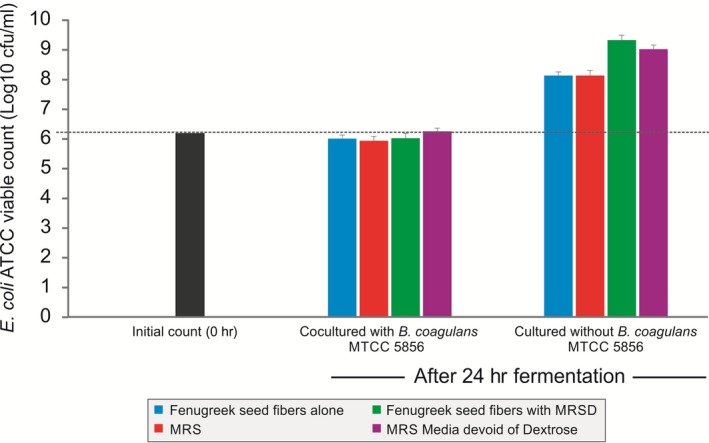
The effect of *Bacillus coagulans* MTCC 5856 on the viability of *Escherichia coli* ATCC 25922 from galactomannan from fenugreek seeds alone and along with MRSD (MRS media devoid of dextrose), MRS, and MRS media devoid of dextrose. Values are average mean of triplicate performed at two different occasions and represented in log_10_ cfu/ml

The effect of *B. coagulans* MTCC 5856 on the growth of *E. coli* ATCC 25922 was determined while fermenting galactomannan from fenugreek seeds. Further, *E. coli* ATCC 25922 was also able to grow in the galactomannan from fenugreek seeds but lesser compared to dextrose group indicating limited utilization of galactomannan from fenugreek seeds (Figure 7). *Bacillus coagulans* MTCC 5856 inhibited growth of *E. coli* ATCC 25922 while fermenting galactomannan and dextrose (Figure 7).

## 4 DISCUSSION

In the current study, we report the prebiotic potential of galactomannan extracted from fenugreek seed. Galactomannan was the major constituent (63.52%) of fenugreek seeds in the form of soluble dietary fibers. One of the basic criteria for the evaluation of fibers as prebiotics is the nondigestibility to gastric acids and to the mammalian digestive enzymes (Gibson & Roberfroid, 1995). An ideal prebiotic must be resistant to upper gastrointestinal tract, therefore, made available to the colonic bacteria as a substrate for the fermentation (Gibson & Roberfroid, 1995). We report the in vitro gastric acid and pancreatic enzymatic nondigestibility of galactomannan which validated the prebiotic nature of galactomannan.

Over the recent years, the concept of functional food has evolved toward the development of dietary supplementation that may alter the gut microbial composition and activities. The scientific rationale behind this concept grows from the current understanding on gut microbiome that the human colon contains health‐promoting species (probiotics), pathogenic organisms, and benign (He & Shi, 2017). Further, gut microbiota in the colon plays a very significant nutritional role by involving major metabolic activities (He & Shi, 2017). Thus, dietary supplementation containing fermentable fiber is a viable route by which the large gut microbiota composition and activities can be modulated. Hence, the utilization of a prebiotic fiber by the clinically efficacious probiotic bacteria would be an ideal approach for dietary supplementation to modulate gut microbiota composition and activities. In vitro fermentation of galactomannan by the probiotic strain *B. coagulans* MTCC 5856 indicated that it is a fermentable carbohydrate which may attribute to an increase in the count of probiotics in colon. Galactomannan utilization by the probiotic strain *B. coagulans* MTCC 5856 was concentration dependent, and a significant utilization (71.4%) was noticed (Figure 5). This is the first time that the utilization of galactomannan from fenugreek by probiotic bacteria *B. coagulans* MTCC 5856 has been reported, and the results demonstrate that *B. coagulans* grew very well by utilizing galactomannan as sole nutritional source and also as a carbon source.

It is well‐known fact that the probiotics proliferate in the gut by fermenting indigestible carbohydrates (fibers) from food. The result of this probiotic fermentation leads to the production of SCFAs in the gut, predominantly acetic, propionic and butyric acids (He & Shi, 2017). Likewise, in our study, SCFAs (acetic, propionic and butyric acids) were produced when galactomannan was fermented by the probiotic strain *B. coagulans* MTCC 5856. Acetic acid was the highest SCFAs among others produced by the *B. coagulans* MTCC 5856 while fermenting the galactomannan. However, significant production of propionic and butyric acids was also observed during the galactomannan fermentation by the *B. coagulans* MTCC 5856. The production of short‐chain fatty acids and lactate as end products of probiotics fermentation of polysaccharides and oligosaccharides was reported previously (Pan, Chen, Wu, Tang, & Zhao, 2009). However, we report for the first time the production of short‐chain fatty acids production as end products of *B. coagulans* MTCC 5856 fermentation of galactomannan from fenugreek seeds. Furthermore, the literature suggests that these short‐chain fatty acids play a pivotal role in the maintenance of overall gut health, intestinal morphology, and function (Fung et al., 2011; Ruemmele et al., 2003). Acetic and propionic acids have been reported to induce apoptosis in human colorectal carcinoma cell lines through the loss of mitochondrial transmembrane potential (Hosseini, Grootaert, Verstraete, & Van de Wiele, 2011). Butyric acid is considered to be a preferred energy source for colonocytes and also lower the luminal pH (Raman et al., 2013). It also has been linked to play an important role in cell cycle processes such as proliferation, differentiation, and apoptosis (Waldecker, Kautenburger, Daumann, Busch, & Schrenk, 2008). Butyrate produced during the gut microbes fermentation of dietary fibers has been reported for neuroprotective effects and also considered to be a significant potential agent as a therapeutic for the brain, both in its dietary and pharmacologic form (Bourassaa, Alima, Bultman, & Ratana, 2016).

Prebiotics have been reported to change the composition of gut flora (microbiota) by suppressing the pathogenic organisms (clostridia and *E. coli*) count and increasing the count of beneficial microbes (bifidobacteria and lactic acid bacteria) (Costalos, Kapiki, Apostolou, & Papathoma, 2008). This change in the gut microbiota composition has been linked with various metabolic syndromes such as obesity, increased plasma glucose levels, hyperlipidemia, hypertension, cardiovascular diseases, and type 2 diabetes mellitus (He & Shi, 2017). In the same way, we observed that the growth of *E. coli* was inhibited when *B. coagulans* MTCC 5856 was grown together in galactomannan indicating selective growth promotion of *B. coagulans* MTCC 5856 and suppression of *E. coli* ATCC 25922. The inhibitory action of *B. coagulans* MTCC 5856 on *E. coli* ATCC 25922 may be due to the production of short‐chain fatty acids (SCFA), lactic acid, and other antimicrobial compounds as well as the competitive nature of probiotic bacteria for nutrients and physical attachment sites. Similar to our finding, the literature suggested that other probiotics have shown the pathogenic bacteria inhibition either by producing organic acids specifically SCFAs and the competitive utilization of nutrients or may be due to physical attachment sites (Callaway, Edrington, Harvey, Anderson, & Nisbet, 2012). Moreover, probiotics have also been reported to activate and enhance the host immune system, which subsequently suppresses the growth of potentially pathogenic bacteria (Hardy, Harris, Lyon, Beal, & Foey, 2013).

## 5 CONCLUSION

Galactomannan from fenugreek seed showed prebiotic potential and was found to serve as a nutritional source as well as carbon source for enhancing the growth of probiotic strain *B. coagulans* MTCC 5856. The combination of galactomannan from fenugreek seed and the probiotic strain *B. coagulans* MTCC 5856 holds a potential for an ideal synbiotic product. However, the prebiotic potential of galactomannan from fenugreek seed against other probiotic organisms needs further investigation.

## CONFLICT OF INTEREST

Authors are employees of Sami Labs Limited/Sabinsa Corporation that manufacture and market Fenumannan^®^ and LactoSpore^®^.

## ETHICAL REVIEW

This study does not involve any human or animal testing.
